# Genetic Code Expansion and Click-Chemistry Labeling to Visualize GABA-A Receptors by Super-Resolution Microscopy

**DOI:** 10.3389/fnsyn.2021.727406

**Published:** 2021-11-26

**Authors:** Alexander Kuhlemann, Gerti Beliu, Dieter Janzen, Enrica Maria Petrini, Danush Taban, Dominic A. Helmerich, Sören Doose, Martina Bruno, Andrea Barberis, Carmen Villmann, Markus Sauer, Christian Werner

**Affiliations:** ^1^Department of Biotechnology and Biophysics, University of Würzburg, Biocenter, Würzburg, Germany; ^2^Rudolf Virchow Center for Integrative and Translational Bioimaging, University of Wuerzburg, Würzburg, Germany; ^3^Institute of Clinical Neurobiology, University of Würzburg, Würzburg, Germany; ^4^Neuroscience and Brain Technologies Department, Istituto Italiano Di Tecnologia, Genova, Italy

**Keywords:** super-resolution microscopy (SRM), click-chemistry, *d*STORM, GABA-A receptor, genetic code expansion

## Abstract

Fluorescence labeling of difficult to access protein sites, e.g., in confined compartments, requires small fluorescent labels that can be covalently tethered at well-defined positions with high efficiency. Here, we report site-specific labeling of the extracellular domain of γ-aminobutyric acid type A (GABA-A) receptor subunits by genetic code expansion (GCE) with unnatural amino acids (ncAA) combined with bioorthogonal click-chemistry labeling with tetrazine dyes in HEK-293-T cells and primary cultured neurons. After optimization of GABA-A receptor expression and labeling efficiency, most effective variants were selected for super-resolution microscopy and functionality testing by whole-cell patch clamp. Our results show that GCE with ncAA and bioorthogonal click labeling with small tetrazine dyes represents a versatile method for highly efficient site-specific fluorescence labeling of proteins in a crowded environment, e.g., extracellular protein domains in confined compartments such as the synaptic cleft.

## Introduction

In the central nervous system, phasic and tonic inhibition is dominantly controlled by γ-aminobutyric acid type A (GABA-A) receptors. These hetero-pentameric, ligand-gated, ionotropic receptors can arrange in different subunit compositions from 19 different subunits to allow high diversity of gating properties, pharmacology, and expression patterns in both specific brain regions and distinct subcellular domains (Wisden and Seeburg, 1992; Barnard et al., 1998; Mody and Pearce, 2004; Olsen and Sieghart, 2008, 2009). Defective GABA-A receptor-mediated inhibition can result in neurodevelopmental and neuropsychiatric disorders, Alzheimer’s disease, and stroke (Vien et al., 2015; de Jonge et al., 2017; Govindpani et al., 2017; Ali Rodriguez et al., 2018; Wang et al., 2018). Surface GABA-A receptors laterally diffuse between synaptic and extrasynaptic sites, an activity-dependent feature that depends on receptor interaction with inhibitory scaffolding molecules like gephyrin (Petrini et al., 2014). So far, super-resolution microscopy of GABA-A receptor subcellular distributions relied on immunolabeling or genetic fluorophore fusions (de Luca et al., 2017; Crosby et al., 2019). However, fluorescent proteins exhibit a size of ∼5 nm and can impede native receptor function. Antibodies can induce crosslinking and internalization and due to their larger size of ∼10 nm introduce a substantial linkage error and impact diffusion of receptors (Weber et al., 1978; Schnell et al., 2012; Dalmau et al., 2017). Linkage error describes the distance between the fluorophore and the binding site of the targeted protein and is calculated by adding the distances of handles needed for the respective labeling procedure. Furthermore, efficient labeling of proteins in a crowded environment or at sterically demanding sites, e.g., in postsynaptic compartments of neurons or at the basal plasma membrane of adherent cells remains challenging because of reduced epitope accessibility (Gray, 1969; Lee et al., 2017; Waldchen et al., 2020). In addition, larger fluorescent labels can likely affect the mobility of proteins, e.g., receptors inside narrow synaptic clefts of 20–30 nm (Beghein and Gettemans, 2017; de Luca et al., 2017).

To reduce the size and linkage error, smaller fluorescent labels such as nanobodies, affimers, aptamers, genetically encoded tags, and superbinding peptides have been developed (Opazo et al., 2012; Nikic et al., 2015; Maric et al., 2017; Thorn, 2017; Schlichthaerle et al., 2018). However, the most direct and less-invasive method to label a protein of interest at a well-defined position with a small fluorophore uses genetic code expansion (GCE) by incorporation of unnatural amino acids (ncAA) that can be labeled by a strain-promoted inverse electron demanding Diels Alder cycloaddition (SPIEDAC) reaction with tetrazine dyes (Beliu et al., 2019). GCE and bioorthogonal click labeling with tetrazine dyes have been used advantageously for site-specific labeling, FRET imaging, and high-end fluorescence imaging of extra- and intracellular protein targets in different model organisms (Ramil and Lin, 2013; Chin, 2014; Uttamapinant et al., 2015; Neubert et al., 2018; Wang et al., 2018; Waterhouse et al., 2018; Serfling et al., 2019; Nikic-Spiegel, 2020; Beliu et al., 2021; Liauw et al., 2021). Although there are reports on the integration of light-activatable potassium channels in rat hippocampal networks *in vivo* and implementation of GCE in living mouse brain was also reported recently, the successful fusion of this method with click chemistry labeling and super-resolution fluorescence microscopy of synaptic receptors in neurons remain challenging (Kang et al., 2013; Ernst et al., 2016). In our recent report, we already could provide evidence that click-chemistry labeling of GCE modified NR1 subunits of the NMDA receptor complex yields functional receptors and can outperform antibody binding in sterically demanding environments (Neubert et al., 2018). In a following report, super-resolution imaging was applied to GCE modified AMPA receptor auxiliary subunits to address masked epitopes in primary neurons and organotypic brain slices (Bessa-Neto et al., 2021). However, a validation of click-chemistry labeling of GCE modified receptor subunits forming multimeric membrane receptors in primary neurons is still missing.

Here, we set out to introduce ncAA in extracellular domains of GABA-A receptor subunits by GCE in HEK-293-T cells and primary cultured neurons followed by site-specific labeling with tetrazine dyes and visualization by *direct* stochastic optical reconstruction microscopy (*d*STORM) and structured illumination microscopy (SIM) (Gustafsson, 2000; Heilemann et al., 2008; van de Linde et al., 2011).

## Results

**FIGURE 1:**
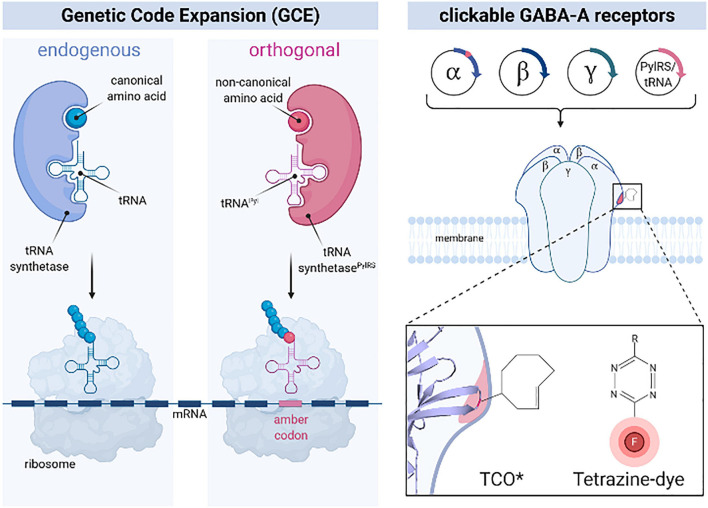
Principle of genetic code expansion (GCE) using amber codon suppression for site-directed labeling of GABA-A receptors with tetrazine-dyes. Introduction of an orthogonal tRNA-synthetase/tRNA pair (red) to the endogenous expression machinery (blue) of mammalian cells allows site-specific incorporation of ncAA in α2 subunits of GABA-A receptors during translation, resulting in full-length GABA-A receptors equipped with a clickable ncAA (here: TCO*) (left). The mutation site is located in the extracellular domain (ATD) of GABA-A receptor α2 subunit (right) and labeled by bioorthogonal click chemistry with a tetrazine-dye (inset) to visualize functional, pentameric GABA-A receptors with unprecedented small linkage error in primary neurons (right).

**FIGURE 2:**
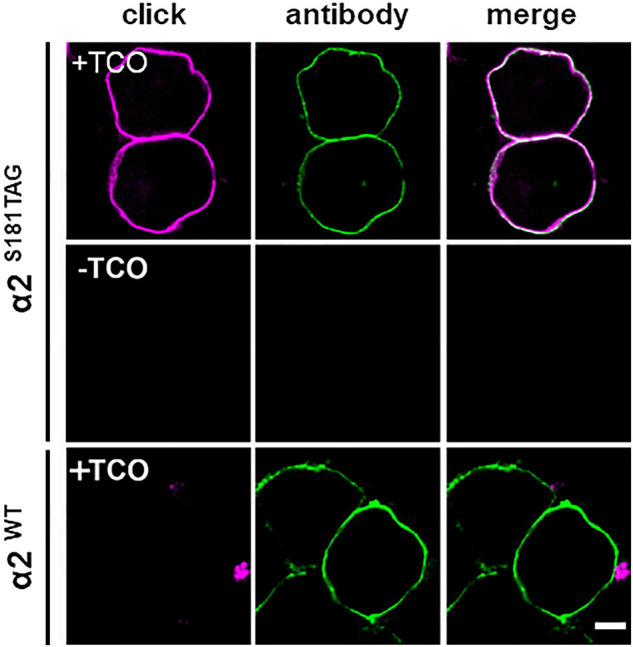
Specificity of bioorthogonal labeling of α2 click-mutant S181TAG of GABA-A receptor α2 subunits. Confocal microscopy of membrane-targeted GABA-A receptor α2 subunits in HEK-293-T cells with coexpression of β1 and γ2 subunits to ensure bonafide membrane delivery of receptors. The ncAA TCO* was click-labeled with the tetrazine-dye H-tet-Cy5 (magenta). Antibody labeling of HA-tag (green) serves as positive control for GCE as the HA tag is located at the n-terminal part of the protein and expression of HA-tag is only feasible after successful ncAA incorporation. The lower panels show negative controls with omission of ncAA feeding (-TCO) after transfection of the S181TAG mutant and low unspecific background signal of H-tet-Cy5 (magenta) on WT α2 receptors (green) supplied with ncAA (+TCO). Scale bar, 5 μm.

We first introduced the AMBER stop codon (TAG) by exchanging lysine or serine codons at different unstructured regions of the extracellular domain of the GABA-A receptor α2 subunit for site-specific labeling (Figure 1 and Supplementary Figure 1). Each Amber mutant construct of GABA-A receptor α2 subunit was co-transfected with β1 and γ2 subunits in HEK-293-T cells to ensure proper surface delivery of α2 subunits, along with an orthogonal tRNA/tRNA-synthetase pair (Nikic et al., 2016). Immediately after transfection, the clickable ncAA trans-Cyclooct-2-en-L-lysine (TCO^∗^) was fed to cells for ncAA incorporation. After bioorthogonal click-labeling of TCO^∗^ with the tetrazine-dye H-tet-Cy5, the labeling efficiency of the different mutants was evaluated by confocal laser scanning microscopy in HEK-293-T cells. Here, Amber mutants S181TAG and S201TAG showed the most efficient labeling demonstrated by a continuous fluorescence signal visible along the cell membrane (Figure 2 and Supplementary Figure 2).

To verify signal specificity, Amber mutants without supplied TCO^∗^ were also investigated but showed negligible staining after the addition of H-tet-Cy5 (Figure 2, middle). Furthermore, α2 wildtype (WT) receptors carrying an HA tag showed neglectable non-specific H-tet-Cy5 signal (Figure 2, lower panel). With a similar approach, we also modified conserved regions of α1 subunits of GABA-A R by GCE. Fluorescence imaging showed that the S181TAG mutant of the α1 subunits can be efficiently labeled with H-Tet-Cy5. In parallel, negative controls demonstrated the specificity of bioorthogonal click-labeling of α1 subunits (Supplementary Figure 3).

**FIGURE 3:**
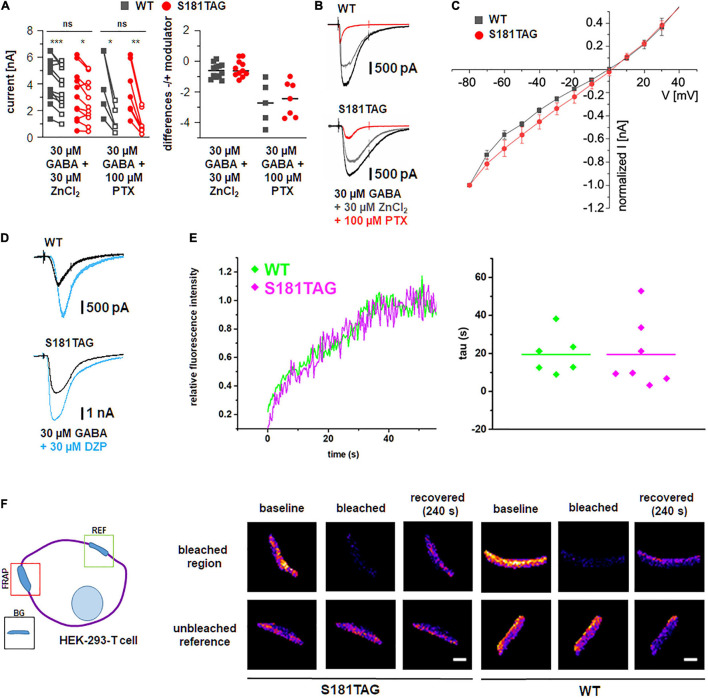
Physiological channel function and diffusion behavior of surface GCE modified GABA-A receptors. **(A)** Validation of physiological function of modified GABA-A receptors by patch-clamp electrophysiology of HEK-293 cells. Left: Amplitude of GABA-evoked currents elicited from WT (gray) and click receptors (red) in control conditions (filled symbol) and upon the application of Zn^2+^ or picrotoxinin (PTX, open symbol). Each pair of symbols represents one patched cell. The number of recorded cells varied between 5 and 12 (*n* = 5–12). Significance level *p ≤ 0.05, **p ≤ 0.01, and ***p ≤ 0.001. Right: Differences between each current pair shown left. **(B)** Representative current traces for data shown in **(A)**. Black traces: 30 μM GABA; gray traces: 30 μM GABA + 30 μM ZnCl_2_; red traces: 30 μM GABA + 100 μM (PTX). **(C)** Comparison of IV curves of WT and click mutant S181TAG applying voltage steps of 10 mV starting at –80 mV. S181TAG modified α2 subunits coexpressed with β1 and γ2 subunits show similar *I*–*V* curves compared to WT receptors. **(D)** Representative current traces of diazepam (DZP) modulation from WT and click receptors. Black traces: 3 μM GABA (WT), 30 μM GABA (S181TAG); blue traces: 3 μM GABA + 3 μM DZP (WT), 30 μM GABA + 30 μM DZP (S181TAG). **(E)** Diffusion behavior of click-mutant S181TAG vs WT in HEK-293-T cell membranes. Impact of amber mutation S181TAG on GABA-A receptor diffusion determined by FRAP experiments. HEK-293-T cells expressing WT (green) and click S181TAG (magenta) GABA-A receptor α2 subunits labeled with anti-HA AF488. Both constructs show similar fluorescence recovery after photo bleaching of selected regions on equatorial membrane. Right: Evaluation of time constant tau (s), defined as the diffusion time necessary to reach 50% of the fluorescence intensity of the recovered state, shows similar values for click-mutant and the WT (WT: 19.50 ± 4.38 vs. S181TAG: 19.51 ± 6.78, mean ± SEM; *p* = 0.617, Mann–Whitney *U* test). This might indicate that the amber mutant is not hindering the diffusion of functional GABA-A receptors. Lines denote mean and single data points are depicted. **(F)** Scheme for FRAP recordings. FRAP recordings taken from selected regions of interest (blue) on anti-HA immunolabeled HEK293-T cell membranes (magenta). Reference regions (REF) on cell membrane without strong photobleaching and background signal (BG) from extracellular signal entering the equation. Exemplary FRAP images of S181TAG mutant and WT labeled with anti-HA AlexaFluor 488 antibodies. Images display baseline, photobleached, and recovered signal after 240 s inside respective regions of interest for photobleached (FRAP) and reference (REF) regions. Scale bar = 1 μm.

Next, we performed patch-clamp experiments in HEK-293 cells expressing the mutant α2 S181TAG, which showed the highest labeling efficiency on the equatorial cell membrane (Figure 2), to demonstrate that the chosen ncAA incorporation site preserves physiological GABA-A receptor function (Figure 3).

Here, patch-clamp recordings revealed similar current amplitudes and *I*–*V* curves for the S181TAG mutant and α2 WT indicating native receptor function (Figures 3A–D). This was further confirmed by the similar block exerted by Zn^2+^-ions (WT 4.3 ± 0.47 nA at 30 μM GABA and 3.7 ± 0.46 nA for GABA + Zn^2+^; *p* = 0.0009; mutant 3.2 ± 0.6 nA for GABA and 2.7 ± 0.5 nA for GABA + Zn^2+^, *p* = 0.0134, two-tailed paired *t-*test) and the comparable sensitivity of currents to picrotoxinin (WT 4.3 ± 0.47 nA at 30 μM GABA and 1.3 ± 0.35 nA for GABA+picrotoxinin, *p* = 0.0124; mutant 2.7 ± 0.5 nA for GABA and 1.1 ± 0.3 nA for GABA+picrotoxinin, *p* = 0.0013, two-tailed paired *t-*test) obtained for α2β1γ2 WT and the mutant α2S181TAG combined with β1γ2. Note there were no significant differences when α2β1γ2 WT was compared to the mutant S181TAG co-transfected with β1γ2 (*p* = 0.157 for GABA, *p* = 0.168 for GABA + Zn^2+^, *p* = 0.644 for GABA + picrotoxinin; unpaired *t*-test; Figure 3A), revealing that functional γ subunit-containing α2 WT and S181TAG GABA-A receptors are in the receptor complex and expressed at the surface membrane of HEK293 cells with similar efficiency. Co-application of GABA and the positive allosteric modulator diazepam demonstrated increased current responses, indicating that the mutation S181 does not affect the benzodiazepine binding site located at the α/γ interface (Figure 3D). The residue S181 is localized in the β8–β9 loop, which has been suggested to enable structural transitions following ligand binding into ion channel opening (Du et al., 2015). As these structural transitions are a prerequisite for ion channel opening and subsequent picrotoxinin binding, the mutation does not affect the overall β8–β9 loop flexibility and thus ion channel function of the mutated GABA-A receptor complex, respectively.

Next, we investigated the influence of ncAA incorporation and click-labeling on the diffusion of receptors by fluorescence recovery after photobleaching (FRAP) experiments with GABA-A α2 wt subunit and the click-labeled S181TAG mutant, carrying HA upstream of the GABA-A α2 sequence. FRAP analysis revealed similar fluorescence recovery times on equatorial membranes of HEK-293T cells for both labeling methods demonstrating the negligible impact of ncAA insertion on receptor trafficking and lateral diffusion (Figure 3E).

**FIGURE 4:**
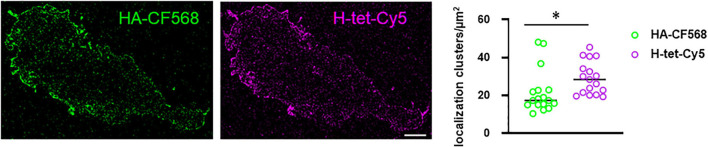
*d*STORM images of immunolabeled HA-tag modified (HA-CF568) and click-chemistry labeled S181TAG GABA-A receptor α2 subunits (H-tet-Cy5) in the same HEK-293-T cell. Cluster analysis applying a DBSCAN algorithm demonstrates that H-tet-Cy5 labeling (magenta) exhibits a higher labeling density (localization clusters/μm^2^) than immunolabeling with anti-HA-CF568 antibodies (green); lines represent median, *p* = 0.005 (*n* = 17, non-parametric Mann–Whitney *U* test). Scale bar: 2 μm. Significance level ^∗^*p* ≤ 0.05.

Overall, these data demonstrate that the small footprint of tetrazine dyes combined with the site-specific introduction of ncAAs preserves the functionality of GABA-A receptors. To visualize the distribution of GABA-A receptors in the plasma membrane of HEK-293-T cells, we used single-molecule sensitive super-resolution imaging by *d*STORM of immunolabeled and tetrazine-dye labeled receptors (S181TAG) in TIRF mode (Haselmann et al., 2018; Siddig et al., 2020). *d*STORM images revealed a homogeneous distribution of GABA-A receptors on the basal plasma membrane independent of the used labeling method. However, DBSCAN cluster analysis (applied to combine repeated localizations from identical fluorophores) detected more localization clusters per μm^2^ for H-Tet-Cy5 click-labeling compared to anti-HA CF568 antibody labeling of the N-terminal HA-tag (Figure 4). The increased number of detected clusters in click labeling compared to conventional antibody staining is fluorophore-independent, since swapping of the fluorescent dyes resulted in similar cluster localizations preserving better labeling efficiency of the click labeling. Moreover, these experiments rule out an effect of photophysical features of the different dye combinations on the detection of clusters (Supplementary Figure 4). Localization clusters result from multiple blinking events of single dyes and dye-labeled antibodies in photoswitching buffer. This result corroborates recent findings that plasma membrane molecules on the basal membrane of adherent cells are more difficult to access by IgG antibodies (Gray, 1969). Thus, GCE with ncAA and bioorthogonal click labeling with substantially smaller tetrazine dyes offers a useful alternative for high-efficient labeling of masked protein epitopes difficult to access by immunolabeling.

**FIGURE 5:**
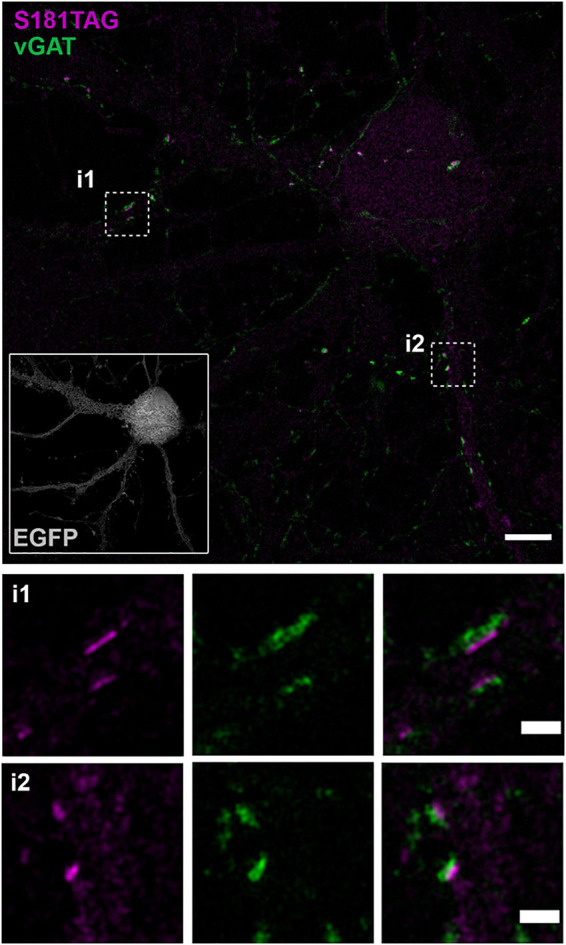
Structured illumination microscopy (SIM) of clickable GABA-A receptor α2 subunits transfected in primary neurons (DIV 14) by effectene. SIM image depicting neuronal morphology by co-expression of cytosolic EGFP (gray, inset, lower left). Postsynaptic S181TAG α2 subunits clicked by Pyrimidyl-tet-ATTO643 (magenta) and immunolabeling of GABAergic presynapses by primary vGAT antibody and goat anti-mouse AF568 secondary antibody (green). Insets provide higher detail images of localization of S181TAG α2 subunits next to presynaptic compartments of GABAergic synapses. Scale bar: 5 μm (overview image), 1 μm (zoomed images).

As chemical synapses represent high-density crowded regions where small fluorescent probes can be used advantageously for tracking and localization of postsynaptic receptors, we tested our approach in cultured primary neurons. We used our S181TAG construct that enables incorporation of the click-α2 subunit along with endogenous GABA-A receptor subunits into complete GABA-A heteropentamers expressed at the neuronal surface. Therefore, we transfected day *in vitro* (DIV) 14 primary neurons with S181TAG and the tRNA/tRNA synthetase pair applying low DNA concentrations and fed neurons with TCO^∗^. After 24 h, we applied Pyrimidyl-Tet-ATTO-643 (Pyr-Tet-ATTO643, Supplementary Figure 5) to target clickable α2 subunits at the surface membrane and concomitantly immunolabeled vesicular GABA transporter (vGAT) to visualize presynaptic GABAergic terminals with a specific primary antibody and secondary goat anti-mouse Alexa Fluor 568 (AF568) labeled antibodies. SIM images of primary neurons revealed specific localization of clickable GABA-A receptors on the neuronal surface that accumulated at synaptic sites juxtaposing the presynaptic vGAT signal demonstrating the successful translation of the method for labeling of postsynaptic receptors (Figure 5).

Of note, the tRNA/tRNA-synthetase plasmid is not optimized for neuronal transfection with lipofection thus leading to low transfection rates. However, the transfection efficiency might be well increased by designing codon-optimized plasmids along with DNA delivery to neurons via nucleofection or AAV and lentivirus-mediated transduction, respectively. Interestingly, the combination of our GCE and click labeling approach with Crispr-Cas mediated knock-in techniques is a promising future perspective for quantification synaptic proteins (Willems et al., 2020). Genetically modified click receptors form functional GABA-A recombinant receptor channels that express at the cell surface and exhibit normal diffusion behavior. Furthermore, when expressed in neurons, genetically modified α2 subunits are incorporated into endogenous receptors and can be targeted to GABAergic synapses. The new GCE labeling approach for GABA-A receptors can be used to unravel nanoscopic changes in receptor distribution at GABAergic synapses during synaptic plasticity. In addition, it might be a valuable tool to study pathological mechanisms in limbic encephalitis associated with GABA-A receptor autoantibodies (Guo et al., 2020). Here, the impact of pathogenic autoantibodies on GABA-A receptor mobility inside and outside of synaptic compartments and the arrangement of receptors at synaptic sites could be documented in pathologic conditions with so far unmatched precision (Spatola et al., 2017).

To conclude, we have shown that GCE with ncAA and bioorthogonal labeling with small tetrazine dyes can be used advantageously for site-specific labeling of difficult-to-access proteins in neurons with minimal linkage error. The method thus provides advantageous labeling of synaptic proteins for super-resolution microscopy and receptor trafficking studies in crowded or sterically demanding nanoenvironments, e.g., small compartments in the synaptic cleft.

## Materials and Methods

### Molecular Biology

The plasmid for expressing the modified α2 subunit of the GABA-A receptor in mammalian cells was obtained from Addgene (Addgene # 49169) (Tretter et al., 2008). The superecliptic pHluorin tag was removed by introducing an XhoI restriction site upstream of the GABA-A coding sequence and subsequent cutting with XhoI-XhoI. The α2-TAG amber stop mutants were produced by introducing a TAG stop codon via PCR-based site-directed mutagenesis of the α2 vector using custom-designed primers (Sigma) and Q5 High-Fidelity DNA Polymerase (New England BioLabs). The plasmid pCMV NES-PylRSAF/tRNAPyl plasmid was a kind gift of Edward Lemke (EMBL, Heidelberg). Plasmids were amplified after transformation to *Escherichia coli* XL1 – Blue followed by MIDI-prep DNA isolation and sequenced (Nucleobond^®^, Xtra Midi, Macherey & Nagel, #740410). GABA-A receptor β1 and γ2 subunits were described previously (Petrini et al., 2011).

### Cell Culture

Labteks were coated with poly-D-lysine (Sigma-Aldrich, #P6407, 0.5 mg/ml) for 1 h at room temperature for adherence of HEK-293-T cells; 1 × 10^5^ HEK-293-T cells were seeded at least 4 h before transfection on four-well Lab-Tek II chambered cover slides (Nunc, cat. no. 155409) and cultured at a 5% CO_2_ atmosphere at 37°C.

HEK-293-T cells (German Collection of Microorganisms and Cell Cultures, Braunschweig, Germany; #ACC635) were maintained in T25-culture flasks (Thermo Fisher, Cat. Nr. 156340) in Dulbecco’s Modified Eagle’s Medium (DMEM, Sigma-Aldrich, #D5796) supplemented with 10% FCS (Sigma-Aldrich, #F7524), and 1% Pen-Strep (Sigma-Aldrich, #P4333).

All experiments with primary neuronal cultures carried out in accordance with the guidelines established by the European Communities Council (Directive 2010/63/EU of September 22, 2010) were permitted by the Italian Ministry of Health and followed the rules approved by the Italian Institute of Technology. Primary cultures of hippocampal neurons were prepared from P0-P1 C57BL/6J mice as previously published (Polenghi et al., 2020). Neurons were plated at a density of 70 × 10^3^ cells/cm^2^ on poly-D-lysine pre-coated coverslips and kept in Neurobasal-A medium (Thermo Fisher, Italy) supplemented with B-27 (Thermo Fisher, Italy) 2%, Glutamax 1% (Thermo Fisher, Italy), and gentamycin 5 mg/ml (Sigma) at 37°C in 7.4% CO_2_.

### Transfection

At 60–80% confluency HEK-293-T cells were transfected using Jetprime (JetPrime, Polyplus) applying a 2:1 DNA/Jetprime ratio. GABA-A receptor subunits were transfected at the following ratio with a total amount of 1750 ng DNA per well: 500 ng α2 subunit, 500 ng β1 subunit, 250 ng γ2 subunit, and 500 ng pCMV NES-PylRSAF/tRNAPyl.

TCO^∗^ (SiChem, SC-8008, Bremen, Germany) was fed separately (250 μM final), diluted in 1M HEPES (one part of 100 mM TCO-A was added to three parts of 1M HEPES, and added in the corner of a single well of four-well Labtek chamber). After 24 h, the medium was exchanged to fresh cell growth medium. The cells were incubated approx. 48 h before labeling and fixation.

Hippocampal neurons were transfected at DIV 14 with the clickable α2 subunit and pCMV NES-PylRSAF/tRNAPyl in 1:2 ratio along with EGFP using Effectene (#301425, Qiagen, Germany) following the manufacturer’s protocol. The following day (DIV 15) each coverslip was supplemented with TCO 250 μM.

### Labeling

Transfected HEK-293-T cells fed with unnatural amino acids were reacted with 1.5 μM tetrazine dye H-tet-Cy5 (#CLK-015-05, Jena Bioscience, Germany) in cell growth medium for 30 min on ice (60 min for *d*STORM experiments). Alternatively, cells were incubated with an anti-HA Alexa Fluor 488, anti-HA Alexa Fluor 555, and anti-HA Alexa Fluor 647 (A21287, 26183-A555, and 26183-A647, Thermo Scientific, concentration: 2 μg/ml), respectively, for 30 min on ice or anti-HA CF568 antibody (5 μg/ml) for 60 min on ice (*d*STORM experiments) to reveal successful incorporation and membrane targeting of GABA-A receptor subunit α2.

For dual-color dSTORM experiments in Supplementary Figure 4, cells were labeled either with Me-tet-Cy5 (3 μM, Jena Biosciences, CLK-1019-1) along with anti-HA CF568 antibody (5 μg/ml, DOL 3.3) or with Me-tet-CF568 (3 μM) together with anti-HA Cy5 antibody (5 μg/ml, DOL 2.2) for 60 min on ice to reveal successful incorporation and membrane targeting of GABA-A receptor subunit α2. Anti-HA CF568 and anti-HA Cy5 were custom-labeled by reacting unconjugated anti-HA antibody (26183, Thermo Scientific) with fivefold excess of CF568 succinimidyl ester (Sigma, SCJ4600027) or Cy5 succinimidyl ester (GE Healthcare, PA15101) for 3 h at room temperature in 100 mM sodium hydrogen carbonate buffer and subsequent removal of unconjugated CF568 or Cy5 fluorophores using Zeba Spin Desalting Columns (Thermo Scientific) following the manufacturer’s instructions.

Me-tet-CF568 was custom-conjugated by reacting 10-fold molar excess of Me-tetrazine-amine (14 mM, Jena Biosciences, CLK-A138-10) with 100 μg of NHS-CF568 (1.4 mM) in anhydrous dimethylsulfoxide (DMSO) (Invitrogen, D12345), containing 0.1% N,N-diisopropylethylamine (DIPEA) (Sigma, 496219-100ML), respectively. Reactions took place at room temperature overnight. Resulting tetrazine conjugates were purified by HPLC on a Kinetex biphenyl column (150 × 4.6 mm) consisting of 2.6 μm particles at 100-Å pore size (Phenomenex, 00F-4622-E0) with a flow rate of 1 ml/min. Solvent A consisted of 0.1% aqueous formic acid (Merck, 33015-1L-M) and solvent B was 95% acetonitrile (ChemSolute, 2697.2500) in 0.1% aqueous formic acid (Merck, 33015-1L-M). Purification was done using a linear gradient of 0% B to 60% B over 30 min. The resulting elution peaks were collected and dried with a speed-vac consisting of a centrifuge (ThermoFisher, SPD111V), a refrigerated vapor trap (ThermoFisher, RVT400), and a vacuum pump (ThermoFisher, VLP80). The pellets were resuspended in double-distilled water, and the final concentration was determined by UV–VIS absorption spectrometry (Jasco V-650). Prior to fixation in 4% formaldehyde and 0.25% glutaraldehyde for 15 min for *d*STORM imaging cells were washed twice in HBSS. For conventional fluorescence microscopy, 4% formaldehyde was used for cell fixation. Following fixation, cells were again washed three times with PBS. Live imaging of clickable GABA-A receptors was performed in the respective medium.

Transfected neurons, supplemented with TCO^∗^ at DIV 15, were fixed at DIV16 with PFA 4% and probed by immunocytochemistry. After blocking with BSA 1% for 10 min, surface labeling of α2 click subunits was performed by incubating neurons with 0.75 μM Pyr-Tet-ATTO643 (#CLK-101, Jena Bioscience, Germany) for 10 min at 37°C. Next, after generous washing in BSA 1%, in order to immunolabel presynaptic GABAergic terminals, neurons were permeabilized (0.2% Triton X-100 for 10 min) and incubated with primary anti-vGAT antibody (SYSY #131 011) followed by goat anti-mouse AF568 secondary antibody (Thermo Fisher). Coverslips were mounted in DAKO fluorescent mounting medium.

### Confocal Microscopy

Confocal microscopy was performed on an LSM700 (Zeiss) using a 63x/1.4 oil immersion objective. 488, 555, and 641 nm laser lines were used for excitation of anti-HA-AF488, anti-HA-AF555, and anti-HA-AF647 antibodies or H-tet-Cy5, respectively. Acquisitions used a pixel size of 100 nm. For comparisons of applied reagents, experimental settings and image postprocessing procedures were kept constant. Images were processed in FIJI by linear adjustment of brightness and contrast only.

#### Fluorescence Recovery After Photobleaching

Fluorescence recovery after photobleaching was applied on HA-antibody labeled (using anti-HA AF488, Thermo Scientific no.: A21287, applied at a final concentration of 2 μg/ml for 20 min) control and mutant constructs expressed in HEK-293-T cells. Fluorescent signal was bleached by applying maximal laser output of the 641 and 561 nm DPSS laser lines and signal recovery (240 cycles, 300 ms interval, 2 μs dwell time) was fitted in ZEN software with a mono-exponential function to yield t_rel:_ *I*(*t*) = *I*_*i*_−(*I*_*f*_−*I*_0_)*exp(−*t*/*t*_*rel*_).

### Patch-Clamp Electrophysiology

Whole cell patch-clamp analysis was performed with a setup as described recently (Schaefer et al., 2019). Recording pipettes had resistances of 3–4 MΩ. Voltage steps reaching from −80 to +40 mV were applied before agonist application and resulting currents were low-pass filtered at 2.9 kHz, and digitized at a sampling rate of 20 kHz with the software Patchmaster Next (HEKA). Data analysis was performed in OriginPro 2020 (OriginLab).

For electrophysiological characterization of the GABA-A receptors, HEK-293 cells were used (CRL-1573; ATCC). Heterologous expression of the respective receptor construct was verified by HA-tag labeling using an anti-HA AF488 antibody (Thermo Fisher, A-21287, 1 μg per 500 μl cell culture medium) applied for 15 min at room temperature and excited via a Zeiss HXP 120 C lamp coupled into the fluorescence beam path of a Zeiss Axio Observer D1 inverse microscope.

The extracellular solution contained (in mM) 137 NaCl, 5.4 KCl, 1.8 CaCl_2_, 1 MgCl_2_, and 5 HEPES; pH 7.3, adjusted with NaOH; 330 ± 1.5 mOsm/L. To activate the receptor, the extracellular solution was supplemented with 30 μM GABA (Sigma Aldrich, A2129) and applied for 50 ms with a pressure of 1 bar using an Octaflow II applications system (ALA Scientific Instruments). For testing the membrane insertion of γ2 subunit, 30 μM ZnCl_2_ or 30 μM diazepam was co-applied together with 30 μM GABA. The pipette solution was composed of (in mM) 120 CsCl, 20 N(Et)_4_Cl, 1 CaCl_2_, 2 MgCl_2_, 11 EGTA, and 10 HEPES; pH 7.2, adjusted with CsOH; 315 ± 1.5 mOsm/L. GABA-A receptor currents were blocked using 100 μM picrotoxin (Sigma Aldrich, P8390). GraphPad Prism 9.0.0 (GraphPad Software, San Diego, CA, United States) was used to calculate mean values, standard error of the mean, and statistical significance. Two-tailed paired (current pairs) and unpaired (WT vs. S181TAG) *t-*tests were used to estimate significance values with ^∗^*p* < 0.05, ^∗∗^*p* < 0.01, and ^∗∗∗^*p* < 0.001.

### Structured Illumination Microscopy

For SIM, immunolabeled primary hippocampal neurons were embedded in DAKO and recorded on a commercial SIM Zeiss ELYRA S.1 system with a Plan-Apochromat 63x/1.40 oil immersion objective; 488 nm and 561 nm OPSL laser, and a 642 nm diode laser were used as excitation lasers with respective filter sets for GFP, AF568, and Pyrimidyl-tet-ATTO643 fluorophores. Recording of image stacks was performed by applying structured illumination using five rotational and five phase variations and the image was reconstructed in ZEN software (ZEN 2.3, Carl Zeiss Microscopy GmbH, Jena, Germany). Chromatic aberration was corrected in ZEN software by applying affine transformations generated from stacks of embedded TetraspeckTM beads (Z7279, Thermo Fisher Scientific, Waltham, MA, United States). Brightness and contrast of reconstructed images were adjusted linearly in FIJI (Schindelin et al., 2012).

### Spectral Characteristics of Dyes and Turn-On Ratios

H-tet-Cy5 was purchased from Jena Bioscience, Jena, Germany # CLK-015-05. Absorbance and emission spectra of Pyr-tet-ATTO643 were recorded in quartz glass cuvettes using an FP-6500 spectrofluorometer (Jasco). Excitation wavelength was positioned over absorption maxima, and spectra were recorded at constant 25°C stabilized via Peltier thermocouple. Time-dependent fluorescence intensities were measured in quartz glass cuvettes using an FP-6500 spectrofluorometer (Jasco). An increase in relative fluorescence for determining the turn-on ratio was measured after performing a click-reaction in cuvette applying 25 μM TCO^∗^ and 1 μM dye solutions.

### *Direct* Stochastic Optical Reconstruction Microscopy

*Direct* stochastic optical reconstruction microscopy imaging was performed on a customized Olympus IX-71 inverted wide-field fluorescence microscope. CF568 and Cy5 were excited using 558- or 640-nm optically pumped semiconductor lasers (OPSL) applying 4 kW/cm^2^ irradiation intensity (Genesis MX561-500 STM, Genesis MX639-1000 STM, Coherent), passing a laser clean-up filter 567/15 (Semrock), respective 640/10 (Chroma) and focused into an oil-immersion objective (60×, NA 1.45; Olympus). Emission was filtered by a dichroic mirror (FF 410/504/582/669 Brightline, Semrock) and spectrally filtered by a bandpass filter (679/41 BrightLine HC, 607/70 Brightline HC, Semrock). The emitted signal was collected in 15.000 frames at 15 ms exposure time on two electron-multiplying CCD cameras (Andor Ixon DU 897). Additional lenses in the emission path yielded a final pixel size of 129 nm for the camera detecting Cy5 and 131 nm for the camera acquiring CF568. Total internal reflection (TIRF) was applied for imaging of basal HEK cell membranes. Photoswitching buffer was PBS stabilized and contained 100 mM cysteamine hydrochloride (Sigma-Aldrich) adjusted to pH 7.4 by addition of potassium hydroxide. Reconstruction of super-resolution images was performed in Thunderstorm (Ovesny et al., 2014) or rapidSTORM 3.3 (Wolter et al., 2012). Localizations were filtered using a detection threshold of 6500 counts, and drift correction was applied by Thunderstorm’s built-in cross-correlation-based algorithm. Chromatic aberration was corrected in FIJI using the plugin BunwarpJ^1^ (Sorzano et al., 2005) by applying elastic transformations generated from stacks of embedded TetraspeckTM beads (Z7279, Thermo Fisher Scientific, Waltham, MA, United States).

### Cluster Analysis of *Direct* Stochastic Optical Reconstruction Microscopy Data

For cluster analysis, a custom-written python script applying the DBSCAN algorithm on the localization data was used. It identified GABA-A receptor α2 clusters by applying the parameters “epsilon” of 20 nm and “minPoints” of 3. Cluster densities were calculated from the number of clusters detected by DBSCAN divided by the area of the region of interest. Non-parametric Mann–Whitney *U* test was applied in Origin for testing statistical significance.

## Data Availability Statement

The raw data supporting the conclusions of this article will be made available by the authors, without undue reservation.

## Ethics Statement

The animal study was reviewed and approved by Regierung von Unterfranken, Approval # 55.2.2-2532-2-811.

## Author Contributions

AK and GB: investigation (lead), visualization (equal) writing–original draft (equal), and writing–review and editing (equal). DJ: investigation (supporting), visualization (equal), writing–original draft (equal), and writing–review and editing (equal). EP, MB, DT, and DAH: investigation (supporting). SD: investigation (supporting), visualization (supporting), and funding acquisition (supporting). CV and AB: supervision (supporting). MS: conceptualization (supporting), funding acquisition (equal), supervision (equal), visualization (supporting), writing–original draft (supporting), and writing–review and editing (lead). CW: conceptualization (lead), funding acquisition (equal), supervision (equal), investigation (supporting), visualization (supporting), writing–original draft (lead), and writing–review and editing (lead). All authors contributed to the article and approved the submitted version.

## Conflict of Interest

The authors declare that the research was conducted in the absence of any commercial or financial relationships that could be construed as a potential conflict of interest.

## Publisher’s Note

All claims expressed in this article are solely those of the authors and do not necessarily represent those of their affiliated organizations, or those of the publisher, the editors and the reviewers. Any product that may be evaluated in this article, or claim that may be made by its manufacturer, is not guaranteed or endorsed by the publisher.

## Footnotes

1
https://imagej.net/BUnwarpJ

## Supplementary Material

Supplementary Figure 1 Click sites of GABA-A receptor α2 subunit (green) SWISS modeled on α1 subunit template (PDB code: 6HUG). Amber sites (magenta and cyan) were introduced using PCR-based site-directed mutagenesis. The two mutants S181TAG and S201TAG (magenta) showed efficient incorporation of the unnatural amino acid TCO^∗^A, when expressed in HEK-293-T cells and labeled with H-tet-Cy5 (Figure 2). In contrast, the two mutants K73TAG and K274TAG (cyan) showed significantly lower fluorescence labeling.

Supplementary Figure 2 Confocal microscopy of GABA-A receptor S201TAG mutant. Top row: α2 click-mutant S201TAG shows similar performance as S181TAG for labeling surface α2 GABA-A receptor subunits by click-chemistry labeling. Modified α2 subunits are targeted by H-tet-Cy3, and anti-HA antibody labeling is applied to verify incorporation of TCO^∗^. Negative controls omit ncAA application and show minor signal for both tetrazine and HA-tag labeling (-TCO). Scale bar = 5 μm.

Supplementary Figure 3 Confocal microscopy testing the specific labeling of GABA-A receptor α1 subunits.GABA-A receptor subunits imaged on the equatorial membrane HEK-293-T cells with coexpression of β1 and γ2 subunits to ensure proper surface delivery of receptors using a confocal laser scanning microscope. First row: α1 click-mutant S181TAG shows slightly lower performance compared to S181TAG in α2 subunits, click-chemistry labeling of incorporated ncAA TCO^∗^ using H-tet-Cy5 (magenta) and HA-Tag labeling (green) serving as positive control for incorporation of the unnatural amino acid. Negative controls include omission of the ncAA after transfection of the S181TAG mutant (middle) and barely no H-tet-Cy5 signal (magenta) on WT α2 receptors (green) (bottom). Scale bar = 10 μm.

Supplementary Figure 4 Evaluating effect of fluorophore selection on cluster detection. *d*STORM images of immunolabeled HA-tag modified (HA-Cy5, magenta) and click-chemistry labeled S181TAG GABA-A receptor α2 subunits (tet-CF568, green) on identical HEK-293-T cell membranes. Right, DBSCAN cluster analysis shows similar trends toward higher labeling density using click-chemistry labeling (tet, filled symbols) compared to anti-HA (HA, open symbols) antibody labeling, independent of conjugated fluorophore. Scale bar = 2 μm.

Supplementary Figure 5 Absorption (black), emission (magenta) spectra, and turn-on ratios of Pyr-tet-ATTO643 applied in this study. **(a)** Absorption and corresponding fluorescence spectrum of Pyr-tet-ATTO643. **(b)** Relative fluorescence increase of Pyr-tet-ATTO643 normalized on initial fluorescence before the addition of 25 μM TCO^∗^.
