# Supplementary figures and images for: Genetic Code Expansion and Click-Chemistry Labeling to Visualize GABA-A Receptors by Super-Resolution Microscopy
Source: Front Synaptic Neurosci. 2021 Nov 26;13:727406. doi: 10.3389/fnsyn.2021.727406 (PMC8664562; doi:10.3389/fnsyn.2021.727406)

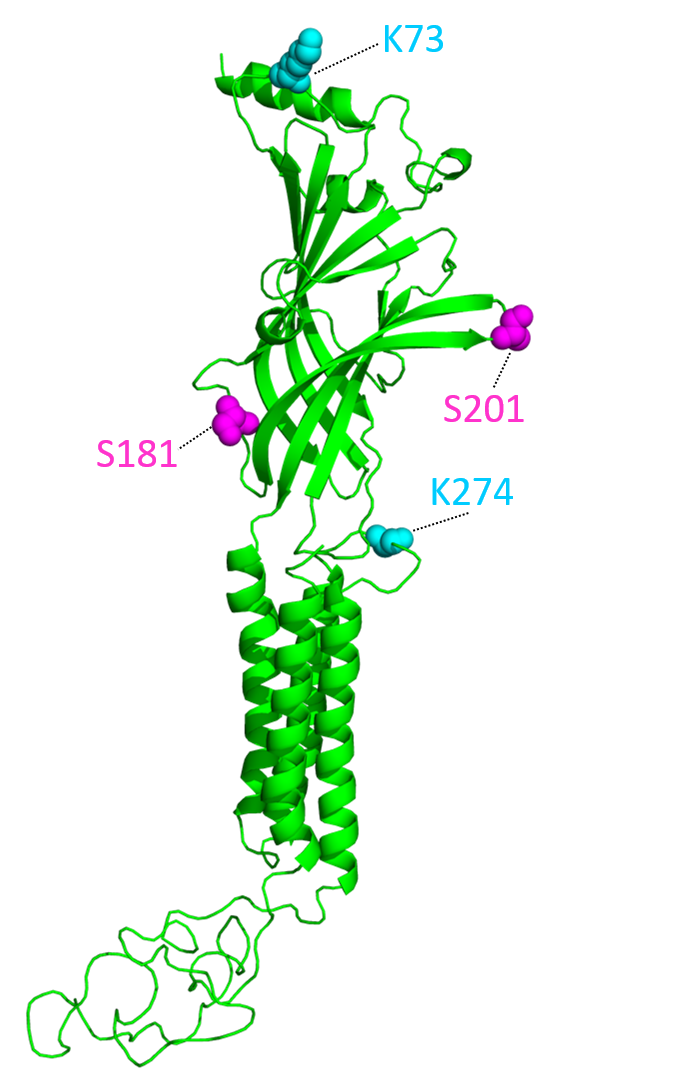

Supplement: Supplementary Figure 1 — Click sites of GABA-A receptor α2 subunit (green) SWISS modeled on α1 subunit template (PDB code: 6HUG). Amber sites (magenta and cyan) were introduced using PCR-based site-directed mutagenesis. The two mutants S181TAG and S201TAG (magenta) showed efficient incorporation of the unnatural amino acid TCO∗A, when expressed in HEK-293-T cells and labeled with H-tet-Cy5 (Figure 2). In contrast, the two mutants K73TAG and K274TAG (cyan) showed significantly lower fluorescence labeling. [file Image_1.TIF]

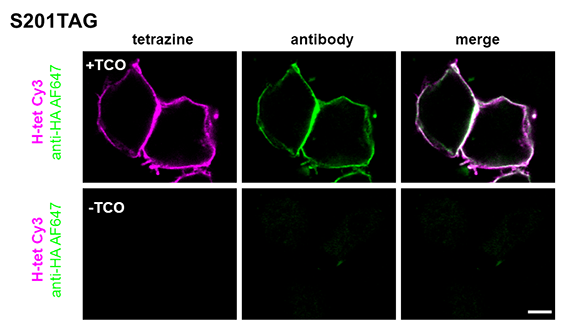

Supplement: Supplementary Figure 2 — Confocal microscopy of GABA-A receptor S201TAG mutant. Top row: α2 click-mutant S201TAG shows similar performance as S181TAG for labeling surface α2 GABA-A receptor subunits by click-chemistry labeling. Modified α2 subunits are targeted by H-tet-Cy3, and anti-HA antibody labeling is applied to verify incorporation of TCO∗. Negative controls omit ncAA application and show minor signal for both tetrazine and HA-tag labeling (-TCO). Scale bar = 5 μm. [file Image_2.TIF]

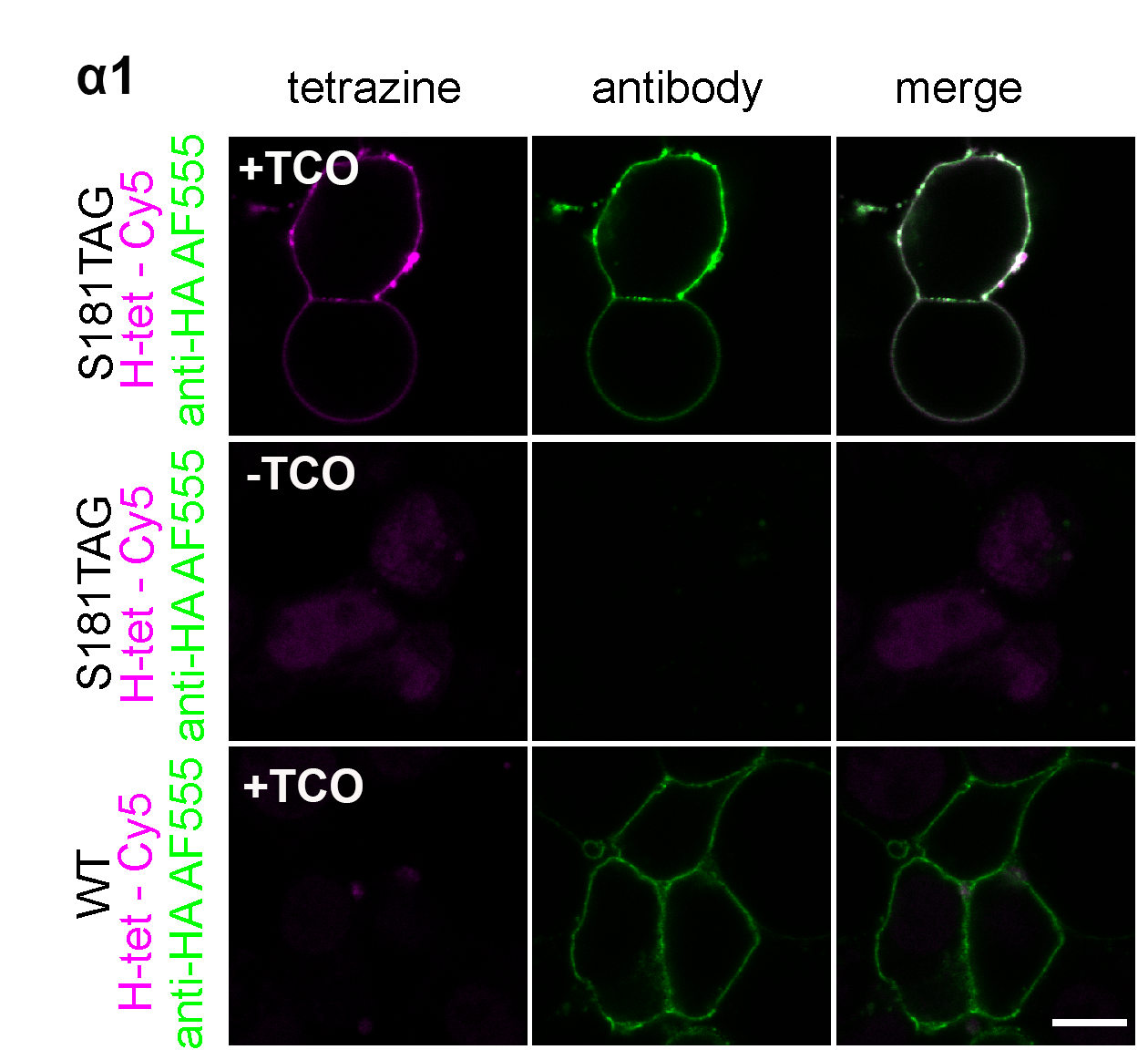

Supplement: Supplementary Figure 3 — Confocal microscopy testing the specific labeling of GABA-A receptor α1 subunits.GABA-A receptor subunits imaged on the equatorial membrane HEK-293-T cells with coexpression of β1 and γ2 subunits to ensure proper surface delivery of receptors using a confocal laser scanning microscope. First row: α1 click-mutant S181TAG shows slightly lower performance compared to S181TAG in α2 subunits, click-chemistry labeling of incorporated ncAA TCO∗ using H-tet-Cy5 (magenta) and HA-Tag labeling (green) serving as positive control for incorporation of the unnatural amino acid. Negative controls include omission of the ncAA after transfection of the S181TAG mutant (middle) and barely no H-tet-Cy5 signal (magenta) on WT α2 receptors (green) (bottom). Scale bar = 10 μm. [file Image_3.TIF]

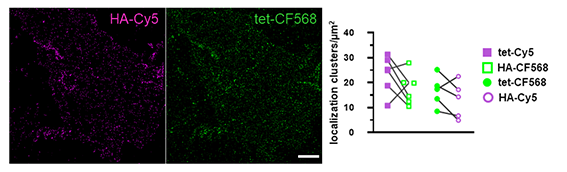

Supplement: Supplementary Figure 4 — Evaluating effect of fluorophore selection on cluster detection. dSTORM images of immunolabeled HA-tag modified (HA-Cy5, magenta) and click-chemistry labeled S181TAG GABA-A receptor α2 subunits (tet-CF568, green) on identical HEK-293-T cell membranes. Right, DBSCAN cluster analysis shows similar trends toward higher labeling density using click-chemistry labeling (tet, filled symbols) compared to anti-HA (HA, open symbols) antibody labeling, independent of conjugated fluorophore. Scale bar = 2 μm. [file Image_4.TIF]

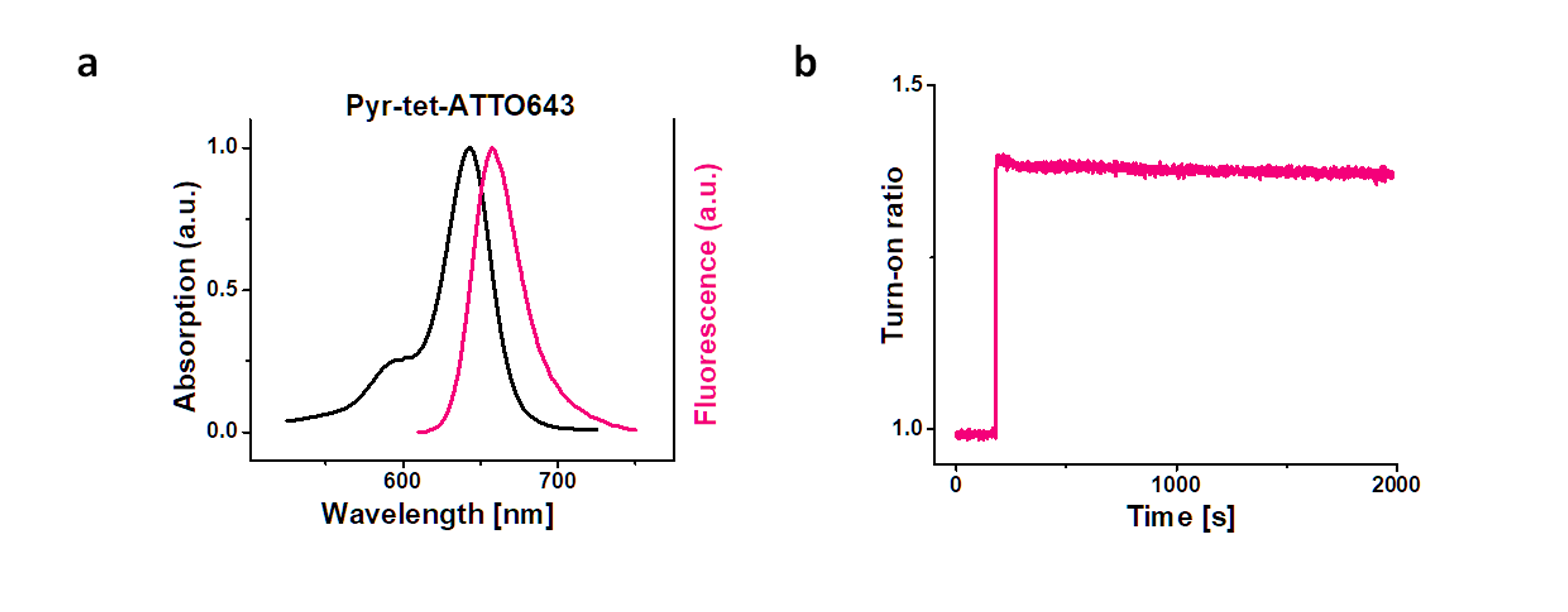

Supplement: Supplementary Figure 5 — Absorption (black), emission (magenta) spectra, and turn-on ratios of Pyr-tet-ATTO643 applied in this study. (a) Absorption and corresponding fluorescence spectrum of Pyr-tet-ATTO643. (b) Relative fluorescence increase of Pyr-tet-ATTO643 normalized on initial fluorescence before the addition of 25 μM TCO∗. [file Image_5.TIF]
